# The Development of Three Questionnaires to Assess Beliefs about Green Exercise

**DOI:** 10.3390/ijerph14101172

**Published:** 2017-10-04

**Authors:** Elliott P. Flowers, Paul Freeman, Valerie F. Gladwell

**Affiliations:** School of Sport, Rehabilitation and Exercise Sciences, University of Essex, Wivenhoe Park, Colchester, Essex CO4 3SQ, UK; pfreeman@essex.ac.uk (P.F.); vglad@essex.ac.uk (V.F.G.)

**Keywords:** attitudes, subjective norms, perceived behavioural control, intentions, theory of planned behaviour, confirmatory factor analysis, physical activity, outdoor recreation

## Abstract

Green exercise is physical activity that takes place in the presence of natural environments. Despite the promising evidence of the benefits, little is known about how individuals’ thoughts and feelings influence participation in green exercise and subsequent outcomes. The aim of the current research was to develop questionnaires using the Theory of Planned Behaviour as a framework that could both directly and indirectly assess attitudes, subjective norms and perceived behaviour control, along with intention toward green exercise. Confirmatory factor analyses confirmed that the indirect, direct, and intention measures all had good overall model fits when tested on a refinement (*n* = 253) and validation (*n* = 230) sample. The questionnaires will contribute towards helping to better understanding individuals’ beliefs about green exercise, how these influence behaviour, and ultimately to enable the development of effective interventions promoting green exercise.

## 1. Introduction

Worldwide figures suggest that around 20% of males and 27% of females are insufficiently active [1]. This is a concern for both public health and the economy, with conservative estimates suggesting that the global economic cost of inactivity is US$53.8b annually [2]. Recently described as a “miracle cure” [3], regular physical activity (PA) is well known to improve health [4] and prevent ill health [5]. Given the importance of PA, a wealth of literature has sought to identify the determinants of exercise behaviour, with individuals’ beliefs and attitudes found to play a key role [6,7]. Beliefs and attitudes have subsequently been targeted in PA interventions with positive effects observed on exercise behaviour and in turn physical and mental health [8,9]. Beyond the overall value of exercise, however, PA that is performed in the natural environment (termed green exercise) may have enhanced health benefits [10,11,12]. To better understand the role of green exercise for health and well-being and to contribute to the development of green exercise interventions, therefore, it is vital to elucidate individuals’ beliefs about green exercise. This current paper contributes to these issues by developing and providing initial evidence for the psychometric properties of three questionnaires that assess beliefs about green exercise.

Researchers have increasingly examined the role of urban green spaces (designed and maintained with human input such as parks and domestic gardens) and natural green space (naturally occurring with minimal human input) for PA and health. A growing body of evidence suggests that the natural environment encourages behaviour change by facilitating people to be more active [13,14,15,16]. Associations have also been found between local green space and mental well-being [17,18,19]. For example, national studies in the UK and the Netherlands have found links between accessible local green space and PA levels [14,20,21]. Further, evidence collected at urban green spaces suggest that people value green spaces for PA and health more so when they view the features and characteristics favourably [22,23,24,25]. As such, the link between urban green spaces and health is now formally recognised by the World Health Organisation [26].

Within natural environments and urban green spaces, individuals can participate in green exercise purposefully (as active participation) or incidentally (as functional engagement) [10]. An example of functional engagement would be walking through a park because it is the shortest route to your destinations. In contrast, if the park was not the quickest route, but was selected because it allowed you to experience nature along your journey, this would be considered active participation. As such, although the experience of nature (i.e., seeing trees and grass) may be similar, the motivation for purposeful and incidental green exercise is different. However, the motivation to do green exercise has not been widely researched [27,28]. Instead, a key focus in the literature has been on the psychological benefits of exercising within different environments. For example, comparisons have been made between performing the same exercise in indoor versus outdoor environments [29,30,31,32] and in urban versus rural outdoor environments [33,34,35,36]. Evidence indicates that green exercise can elicit psychological improvements over and above that of indoor and urban exercise [15,16,30,37,38,39]. Specifically, acute bouts of green exercise have been shown to facilitate reductions in anxiety [40], reductions in mood disturbance [41,42], and improvements in self-esteem [43,44,45]. Moreover, dose-response relationships have shown that the greatest benefits on mood and self-esteem occur within the first five minutes of green exercise [42].

Despite the promising evidence for the benefits of green exercise, little is known about how individuals’ thoughts and feelings about green exercise influence participation and subsequent outcomes. This is surprising given the myriad of studies that have demonstrated the importance of understanding the beliefs about PA more generally [6,7]. A small number of studies, however, have shown that perceptions of local green space may predict visit frequency more than quantity and proximity [14,46,47,48]. Additionally, the New Ecological Paradigm [49] and Nature Relatedness Scale [50] are tools that have been developed to measure how people feel towards nature, but these have rarely been applied in the context of PA. Nevertheless, one recent study found that nature relatedness was a strong predictor of visit frequency to local green space [14]. An exploration of green exercise beliefs is needed to better understand how to augment engagement with green exercise behaviours.

**Figure 1:**
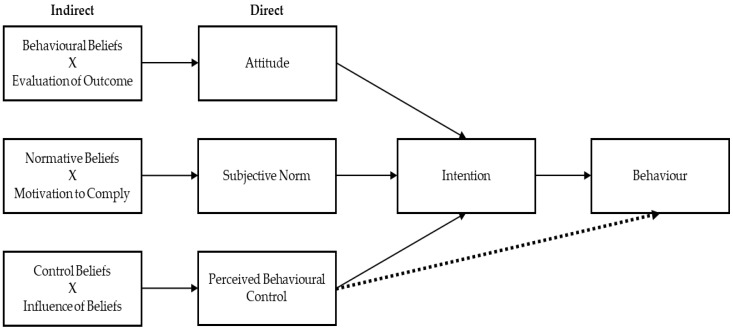
The theory of the planned behaviour (adapted from Ajzen, 2006; [62]).

The theory of planned behaviour (TPB) is an important framework that has been used to advance understanding of how cognitions influence PA behaviours generally [7,51,52]. Derived from the theory of reasoned action [53], the TPB assumes that intention to perform a behaviour is best predicted when individuals evaluate the behaviour positively (attitudes), believe peers will support the behaviour (subjective norm), and perceive the behaviour to be within their capabilities (perceived behavioural control; PBC). TPB factors can be assessed directly (e.g., by asking people to report attitudes, norms, and PBC) or indirectly (e.g., by asking people about specific behavioural beliefs and combining the scores with a paired evaluation of the belief) (see Figure 1). As such, indirect behavioural, normative, and control beliefs combine with evaluations of those beliefs to predict the respective direct measures of attitudes, subjective norms, and PBC. Not only does this enable correlational analyses to establish convergent validity, but also serves to capture the different underlying cognitive processes of each measure [54]. Despite concerns about the intention-behaviour gap, the TPB [55] has been the most successful approach in exercise psychology for predicting participation from beliefs [56]. One meta-analysis revealed that nearly half of variance in PA intentions, and over a quarter of variance in PA behaviours could be explained by beliefs [7].

As a form of PA, some authors have hypothesised that green exercise can be modelled using the TPB [15,57]. Using a systematic review, Calogiuri and Chroni [15] integrated the green exercise literature with the TPB to propose a schematic model of motivational processes underlying the relationship between natural environments and physical activity behaviours. The evidence collected supports using the TPB framework to explore the green exercise phenomenon. Moreover, empirical evidence—collected using ad hoc TPB questionnaires—has shown that beliefs may predict behaviours such as park visitation [58], participation in outdoor recreation programs [59], outdoor walking [60], and outdoor pool use [61]. Although these studies have enriched understanding into the role of beliefs on specific green exercise behaviours, the need to create measurement tools for individual studies does not encourage a proliferation of research into green exercise beliefs and also impairs the ability to synthesise evidence across studies. A valid measure of beliefs about green exercise is necessary to deepen our understanding of the relationship between green exercise and health, understand variations in green exercise beliefs, and develop intervention to increases green exercise.

**Figure 2:**
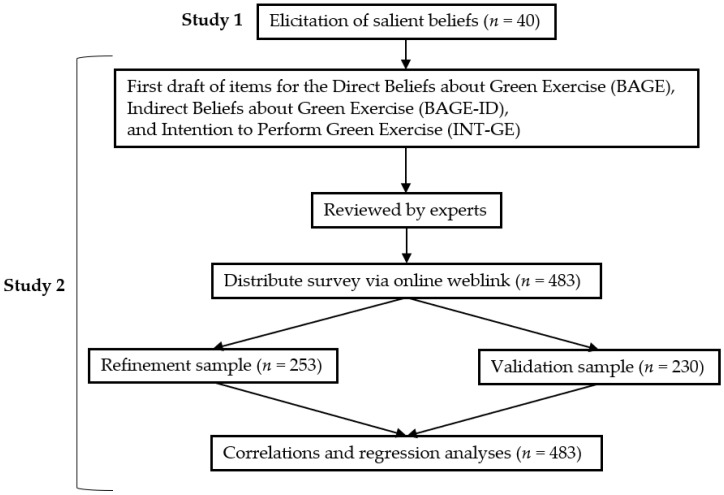
Phases in the construction of the questionnaires.

The aim of the current article was to develop and provide initial evidence of the validity of three questionnaires that assess global beliefs about green exercise amongst the general population. The research drew upon comprehensive guidelines for developing TPB questionnaires [63], which provided recommendations for overall structure, item wording, and scoring criteria, and a recent a systematic review that identified a 16-point criteria for assessing the quality of TPB questionnaires [64]. These criteria emphasise the importance of methodological rigour, such as the inclusion of an elicitation study, developing both indirect and direct measures, and establishing content validity. As such, the current research was divided into two distinct studies (see Figure 2). Study 1 focused on the elicitation and content analysis of salient beliefs about green exercise, and Study 2 focused on the development and validation of the three questionnaires that assess indirect beliefs, direct beliefs, and intention to perform green exercise respectively.

## 2. Study 1

Before creating a TPB questionnaire, an elicitation study is recommended to capture the salient beliefs that individuals hold toward a given behaviour [53,55,64]. Authors suggest that a minimum sample of 25 participants is required to sufficiently ascertain a representation of salient belief amongst a population [63,65]. Content analysis of responses to carefully worded open-ended questions provides the beliefs that underlie the indirect psychological factors of TPB [63]. However, within the PA domain, elicitation studies are not routinely used in TPB research [7,66]. Indeed, out of 150 TPB studies exploring PA, only 47 included a prior elicitation of beliefs [66]. The purpose of Study 1 was to elicit the salient beliefs individuals hold about green exercise.

### 2.1. Method

#### 2.1.1. Participants

The sample comprised 40 adults (22 women, 18 men, mean age 27.1 ± 10.5 years, age range 18–59 years). Participants were undergraduate students (50%), employed (35%), self-employed (7.5%), or other (7.5%).

#### 2.1.2. Measure

In accordance with recommendations [62], questions were specifically worded to elicit beliefs about the (dis)advantages of green exercise and whether it is liked or disliked (behavioural beliefs), who would (dis)approve of green exercise (normative beliefs), and factors that facilitate or impede green exercise behaviours (control beliefs). The questions were preceded by the following statement: “Some people like to spend free-time in local green spaces such as parks, woodlands and sports fields. When people do exercise at these places, we like to call it green exercise. We want to find out what people think about green exercise”. Example questions include: “What do you think are the disadvantages of doing green exercise as part of your weekly physical activity” and “What do you think would make it easy for you to do green exercise as part of your weekly physical activity?”. Each question was followed by five blank lines to allow for multiple responses.

#### 2.1.3. Procedure

The research was conducted in December 2015 following ethical approval from the University of Essex, Ethical Committee (15/BS/403/EF). All participants provided informed consent. A hard copy of the questionnaire was completed by 20 undergraduate students using convenience sampling at University of Essex, Colchester Campus. To reduce order effects, three versions of the questionnaire were created each with a different order of questions. An online version—with randomised question order—was also created using Qualtrics Software (Provo, UT, USA). This was distributed as a short web-link via various social media platforms, such as LinkedIn and Twitter. Data collection was closed once 20 participants had completed the online version and 20 had completed the hard copy version.

#### 2.1.4. Analyses

The lead author conducted line-by-line content analyses to find emerging themes from the responses. Next, all salient beliefs were categorised into the themes and corroborated with a second author. Following recommendations [63], the advantages and disadvantages responses were coupled with the likes and dislikes responses respectively.

### 2.2. Results

Beliefs pertaining to the advantages and likeable features of green exercise were the most commonly reported (*n* = 198, see Table 1). In contrast, only 11 responses were given around whether people would disapprove of them doing green exercise. Within the themes, poor weather was the most commonly cited disadvantage/dislikeable feature of green exercise (*n* = 61; 46%). However, climatic conditions were also reported 19 times as a facilitating factor of green exercise.

Overall, the most prevalent responses which reflected behavioural beliefs were poor weather (*n* = 61), fresh air (*n* = 32) and positive affect (*n* = 31). The most prevalent responses which reflected normative beliefs were family (*n* = 19), friends (*n* = 15), and health professionals approving of green exercise (*n* = 10). Finally, the most prevalent responses relating to control beliefs were weather (*n* = 20), free time (*n* = 20), and access (*n* = 16). There were no discernible differences in the beliefs elicited from the student and general population samples.

## 3. Study 2

The beliefs elicited in Study 1 provided the foundation for the indirect questionnaire in Study 2. According to Oluka, Nie and Sun [64], this is an essential criterion in developing TPB questionnaires. Three separate questionnaires were developed to assess attitudes, subjective norms, and PBC both indirectly and directly, along with intention to perform green exercise: the Indirect Beliefs about Green Exercise Questionnaire (BAGE-ID), the Beliefs about Green Exercise Questionnaire (BAGE), and the Intention to Perform Green Exercise Questionnaire (INT-GE).

### 3.1. Method

#### 3.1.1. Initial Scale Construction

Adhering to TPB principles, themes from the elicitation study were used to inform the development of the items for the BAGE-ID [64]. As per recommendations [63], over 75% of salient beliefs elicited were covered in the questionnaire items. The responses from each elicitation question corresponded directly to a particular TPB factor in the BAGE-ID: responses from the (dis)advantages/(dis)likes questions provided the themes for the behavioural belief items, (dis)approve questions related to normative beliefs, and easy/difficult questions related to control beliefs. Furthermore, each factor in the BAGE-ID contained particular question types: behavioural beliefs consisted of both instrumental and experiential evaluations of green exercise; normative beliefs consisted of injunctive and descriptive evaluations of norms; and control beliefs consisted of self-efficacy and controllability items. As per the guidelines [63], each belief item was paired with an evaluation item that reflected the same theme.

The BAGE and INT-GE were developed using TPB measurement guidelines [62,63]. Items in the BAGE were worded to reflect direct beliefs about attitudes, subjective norms, and PBC towards green exercise. Items in the INT-GE were based on pre-existing phrases from [63]. At least five items (or pairs of items in the BAGE-ID to reflect beliefs and the evaluations of those beliefs) were created by the lead author for each TPB factor. All items were reviewed for wording and relevance by three authors who have previously published peer-reviewed green exercise research. After some minor alterations, the questionnaires were completed by two non-academic professionals who provided external feedback; no further modifications were made. The BAGE-ID, BAGE, and INT-GE consisted of 32 (16 pairs), 16, and 5 items respectively.

#### 3.1.2. Indirect Beliefs about Green Exercise (BAGE-ID)

The 32 items in the BAGE-ID operated in pairs. In the indirect measure of attitudes, six behavioural beliefs (responded to on a 1 to 7 scale) were multiplied with six evaluations of outcomes (responded to on a −3 to +3 scale). Therefore, each pair of items produced a single datum from −21 to +21. For example, the response to “when I do Green Exercise, I feel better about myself afterwards (1) Strongly Agree to (7) Strongly Disagree” was multiplied by the response to “feeling better about myself after Green Exercise is… (−3) Extremely Undesirable to (+3) Extremely Desirable”. For the indirect measure of subjective norm, five normative beliefs (−3 to +3) were multiplied with motivation to comply (1 to 7). For example, “My friends think I should do Green Exercise (−3) Strongly Disagree to (+3) Strongly Agree” was paired with ‘My friends approving of me doing Green Exercise is… (1) Not at all Important to (7) Extremely Important’. For the indirect measure of PBC, five control beliefs (1 to 7) were multiplied with perceived power of beliefs (−3 to +3). For example, “The amount of green space in my local area influences my decision to do Green Exercise (1) Strongly Agree to (7) Strongly Disagree” was paired with “Having more local green space would make me more likely to do Green Exercise (−3) Very Unlikely to (+3) Very Likely”.

#### 3.1.3. Direct Beliefs about Green Exercise (BAGE)

Each of the 16 items in the BAGE had a response scale of 1 to 7. The attitudes factor consisted of six items assessed on a bipolar scale of adjectives. An example item and response scale was: “Doing Green Exercise as part of my weekly physical activity is… (1) Pleasant to (7) Unpleasant”. The subjective norms factor included five items that were all scaled from (1) Strongly Disagree to (7) Strongly Agree. For example, “People often ask me to do Green Exercise with them…”. The PBC factor also had five items; four items were scaled from (1) Strongly Agree to (7) Strongly Disagree, and one item was scaled from (1) Very Difficult to (7) Very Easy. For example, “I am confident I could do Green Exercise if I wanted to… (1) Strongly Disagree to (7) Strongly Agree”.

#### 3.1.4. Intention to Perform Green Exercise (INT-GE)

The INT-GE consisted of five items measured on a 7-point scale; four items were scaled from (1) Strongly Agree to (7) Strongly Disagree, and one item was scaled from (1) Very Unlikely to (7) Very Likely. An example item was “I want to do Green Exercise at least once per week for the next four weeks… (1) Strongly Disagree to (7) Strongly Agree”.

#### 3.1.5. Participants

The total sample comprised 483 adults (306 women, 177 men, mean age 45.4 ± 16.0 years, age range 18–83 years). The majority of the participants were employed (60.5%), with the remaining participants being retired (18.6%), self-employed (8.1%), students (6.8%), or other (6.0%). Additionally, the majority of participants reported their ethnicity as white (95.4%). Over half of participants (52%) reported a household income less than £49,999, 38% reported over £50,000, and 10% reported not knowing or did not wish to say.

For the analyses, the participants were randomly divided into two samples: a refinement sample (*n* = 253) and a validation sample (*n* = 230). There was a significant difference in the age for the refinement (M = 48.6 ± 16.5 years) and validation (M = 41.9 ± 14.9 years) groups, *t* (479) = 4.62, *p* < 0.01. There was no significant difference in gender split (χ^2^ (1) = 2.13, *p* > 0.05) or income (χ^2^ (7) = 4.85, *p* > 0.05).

#### 3.1.6. Procedure

The research was conducted between February and June 2016 following ethical approval from the University of Essex, Ethical Committee (16/BS/420/EF). All participants provided informed consent. An online survey was created using Qualtrics Software (Provo, UT, USA). The survey was primarily distributed as a short web-link via email to contacts on social media via the lead researchers personal accounts, and internet messaging services to professional networks. It was also marketed via two specialist participant recruitment websites: callforparticipants.com and findparticipants.com. On the first, it was placed as a static advert, and on the second it was distributed to 219 individuals who had registered to receive such surveys. Participants provided demographic information before completing all items from BAGE-ID, BAGE, and INT-GE questionnaires. All items within the three questionnaires were randomised to reduce order effects.

#### 3.1.7. Analyses

Initially, data from the refinement and validation samples was screened for non-normality, missing data, and outliers. Screening revealed multivariate non-normality and less than 1% missing data. Missing data was imputed using the regression method available on IBM SPSS 23 (Chicago, IL, USA) and constrained to match questionnaire item response options (i.e., 1 to 7/−3 to +3 in whole numbers). Where appropriate, items were recoded so that higher numbers reflected stronger agreement with the item. Paired items in the BAGE-ID were multiplied.

Confirmatory factor analyses with maximum likelihood estimation were then performed on IBM AMOS 23 (Chicago, IL, USA) to assess the factorial validity of the BAGE-ID, BAGE, and INT-GE in turn. Initially, analyses were conducted on the refinement sample. A three-stage sequential model testing approach was adopted separately with the BAGE-ID and BAGE in the refinement sample [67], whereas a single stage was used for the INT-GE. First, to assess convergent validity, single-factor models of attitudes, subjective norms and PBC were run individually. Overall model fit and individual item indices (described below) were checked and where necessary items were deleted and the models re-examined.

Second, each subscale within a questionnaire was paired in turn with all subscales in that questionnaire and two-factor models were tested. This allowed identification of ambiguous items. Overall fit indices of each model were considered along with modification indices which indicated whether the fit could be improve if items were freed to cross-load on another subscale. Third, all subscales within a questionnaire were included in a three-factor model and model fit and individual item indices were examined. The final models for the BAGE-ID, BAGE, and INT-GE identified using the refinement sample were then re-tested in the validation sample in turn.

Overall model fit was assessed using numerous indices. Following recommendations [68], Bollen–Stine bootstrapping was used to account for non-normality, thus producing a Bollen-Stine chi-squared score (BSχ^2^) for overall model fit. Additionally, the comparative fit index (CFI), Tucker–Lewis index (TLI), and root mean square error of approximation (RMSEA) were also used to examine model fit from three different classes [67,69].

Consistent with recommendations [69,70], scores above 0.95 for the CFI and TLI, and scores below 0.6 for the RMSEA were considered as indicators of good model fit, although these were not applied as “golden rules” [71]. Beyond overall model fit, examination of modification indices, factor loadings, and standardised residuals were screened to help identify poorly fitting items and guide model improvement. Following suggestions [72,73], modification indices above 7, standardised residuals greater than an absolute value of 2, and factor loadings below 0.40 were considered a concern.

Beyond examining the factorial validity of the BAGE-ID, BAGE, and INT-GE, additional analyses were conducted to further assess the psychometric properties of the instruments. First, to account for greater reliability of items with higher weights, composite reliability was calculated using a formula adapted from Fornell and Larcker [74].

Scores above 0.60 were considered acceptable. Using the entire sample (*n* = 483), parallel-form reliability was assessed by using Pearson’s correlation analyses to explore if beliefs obtained indirectly (BAGE-ID) correlated with direct measures of attitudes, subjective norms, and PBC (BAGE). A forced entry regression analysis was also conducted to assess whether the three factors from the BAGE-ID predicted intention (INT-GE). The process was repeated for the BAGE. Statistical significance was accepted at *p* < 0.05 in the correlation and regression analyses.

### 3.2. Results and Discussion

#### 3.2.1. Validation of the Instruments with the Refinement Sample

##### BAGE-ID

The fit statistics and factor loadings at the single-factor stage for the initial BAGE-ID are shown in Table 2. Mixed results were found. All the BSχ^2^ to degrees of freedom ratios were below 2, CFI values were 0.91–0.98, TLI values were 0.82–0.97, and RMSEA values were 0.06–0.09. For the indirect measure of attitudes, all items had reasonable factor loadings (>0.54) and were subsequently retained at this stage. For the indirect measure of subjective norms, the item relating to current or potential employers had a low factor loading (0.15) and was removed. This may be due to a disparity between the employment rate of the sample (68.1%) and the general population (74.5%; [75]). The health professionals item also had a low factor loading but was retained at this stage as the overall model fit was good following deletion of the employer item (CFI = 1.00, TLI = 0.99, and RMSEA = 0.03). For the indirect measure of PBC, items relating to the weather and free time were removed due to low factor loadings (0.15 and −0.08, respectively). As with attitudes and subjective norms, once the items with the lowest factor loadings were removed, the overall model fit improved. Therefore, the individual factors were deemed to have sufficient convergent validity to progress to the paired-factor stage.

At the paired-factor stage, the factors were paired into three models (Attitudes × Subjective Norms, Attitudes × PBC, and Subjective Norms × PBC). All of the paired-factor models had good model fits (Table 3). The BSχ^2^ to degrees of freedom ratios were below 2, CFI values were 0.93–0.98, TLI values were 0.90–0.97, and RMSEA values were 0.04–0.09. As the overall fits were good and no items had particularly poor factor loadings, the factors progressed onto the final model. This included a combination of all three factors: indirect measures of attitudes, subjective norms, and PBC. The full three-factor model had a good model fit (BSχ^2^/df = 1.10, CFI = 0.97 TLI = 0.96, and RMSEA = 0.05), and all factor loadings were above 0.40 (see Table 4). Further, the attitudes factor had good composite reliability (ρc = 0.83), whereas subjective norms and PBC had reasonable composite reliability (both ρc = 0.63).

##### BAGE

The fit statistics and factor loadings at the single-factor stage for the initial BAGE are shown in Table 5. Mixed results were found. All the BSχ^2^ to degrees of freedom ratios were below 2, CFI values were 0.89–0.95, TLI values were 0.77–0.90, and RMSEA values were 0.06–0.14. For attitudes and subjective norms, the individual item with the lowest factor loadings was removed. For attitudes, even though the factor loading of the item relating to green exercise being (un)healthy was reasonable (0.54), as the modification indices (>22) revealed that the chi-squared statistic would improve if the item was removed. The overall model fit subsequently improved. For subjective norms, the item relating to social pressure to do green exercise had the lowest factor loading (0.17) and was subsequently removed. After the removal of the items, the subsequent model fits of the attitudes and subjective norms factors were good (CFI = 0.98–1.00, TLI = 0.96–1.05, and RMSEA = 0.00–0.08).

The PBC factor was more problematic as three items had poor factor loadings (<0.40). The individual item (“whether I do Green Exercise or not is entirely up to me”) with the lowest factor loading was removed in the first instance, which resulted in a good model fit (CFI = 0.98, TLI = 0.94, and RMSEA = 0.04). Two additional items remained a concern, as both “the decision to do Green Exercise is beyond my control” and “I choose when and where I do Green Exercise” had low factor loadings (0.30 and 0.35, respectively). Given the good overall model fit and similar factor loadings, both items were retained at this stage pending further examination at the paired-factor and three-factor stages.

The fit statistics at the two-factor stage are shown in Table 6. All three paired-factor models had good fits: the BSχ^2^ to degrees of freedom ratios were below 2, CFI values were 0.95–0.98, TLI values were 0.92–0.97, and RMSEA values were 0.03–0.05. The factors were therefore progressed to the final three-factor model.

As shown in Table 7, the problematic items in the PBC factor still had poor factor loadings (both 0.27). Further inspection revealed that all modification indices were below 7 and standardised residuals were below 2, and that the full three-factor model had a good model fit (BSχ^2^/df = 1.05, CFI = 0.98, TLI = 0.97 and RMSEA = 0.03), so the items were retained for further examination in the validation sample. This kept the minimum number of items within each TPB factor to three, consistent with the three-indicator rule described by Blunch [76]. The attitudes factor had good reliability (ρc = 0.82), whereas subjective norms and PBC had reasonable reliability (ρc = 0.54 and 0.44, respectively).

##### INT-GE

The fit statistics and factor loadings of the INT-GE are shown in Table 8. The model fit was excellent. The BSχ^2^ to degrees of freedom ratio was below 2, the CFI was 1.00, the TLI was 1.01, and the RMSEA was 0.00. The composite reliability was good (ρc = 0.89).

#### 3.2.2. Analysis of the Validation Sample

The factor structure of the BAGE-ID, BAGE, and INT-GE were further explored in the validation sample. All models had a good fit (BSχ^2^/df = 0.66–1.15, CFI = 0.95–1.00, TLI = 0.91–1.01, and RMSEA = 0.00–0.07). All items in the BAGE-ID, BAGE, and INT-GE had factor loadings above 0.40 (see Table 4, Table 7 and Table 8). The factor loadings of the two problematic direct PBC items from the refinement sample were both higher (0.41 and 0.44) and significant in the validation sample, suggesting that the items should be retained. For the BAGE-ID, each indirect factor had good composite reliability (attitudes ρc = 0.81, subjective norms ρc = 0.70, and PBC ρc = 0.65). Reasonable results were found in the BAGE (attitudes ρc = 0.88, subjective norms ρc = 0.62, and PBC ρc = 0.58). Composite reliability for the INT-GE was good (ρc = 0.86). Overall, the analyses conducted on the validation sample provide additional evidence of the psychometric properties of the BAGE-ID, BAGE, and INT-GE, and the findings suggest that the factor structures and other indices are relatively consistent across the two samples.

#### 3.2.3. Correlation and Regression Analyses of the Full Sample

To provide evidence for parallel-form reliability and the theoretical predictions of the TPB, correlations between the respective subscales of the BAGE-ID and BAGE were examined in the full sample (*n* = 483). The measures of attitudes (*r* = 0.71, *p* < 0.01) and subjective norms (*r* = 0.61, *p* < 0.01) were significantly correlated, but the measures of PBC were not (*r* = −0.01, *p* > 0.05). This may partly be due to the lower factor loadings found within PBC factor of the BAGE in the refinement sample. Further exploration may be warranted to see if the wording of those items could be improved.

Consistent with the predictions of the TPB, linear regressions were run to explore whether attitudes, subjective norms, and PBC predict intention to perform green exercise. First, after controlling for age and gender, the three indirect factors (behavioural, normative, and control beliefs) significantly predicted intention to perform green exercise, *r^2^* = 0.34, *F*(3, 475) = 74.18, *p* < 0.01. All three factors made unique significant contributions: behavioural beliefs (*b* = 0.12, *p* < 0.01, *sr^2^* = 0.23), normative beliefs (*b* = 0.04, *p* < 0.01, *sr^2^* = 0.02), and control beliefs (*b* = 0.03, *p* < 0.05, *sr^2^* = 0.01).

Similarly, the direct factors (attitudes, subjective norms, and PBC) significantly predicted intention to perform green exercise, *r^2^* = 0.51, *F*(3, 475) = 153.34, *p* < 0.01. All three factors made unique significant contributions: attitudes (*b* = 0.70, *p* < 0.01, *sr^2^* = 0.24), subjective norms (*b* = 0.28, *p* < 0.01, *sr^2^* = 0.08), and PBC (*b* = 0.30, *p* < 0.01, *sr^2^* = 0.06). These results provide initial evidence that the factors in the questionnaires are broadly related in a manner consistent with TPB.

## 4. General Discussion

The aim of the current study was to develop and provide initial evidence of the validity of three questionnaires that assess individuals’ beliefs about green exercise. Using the TPB as a theoretical framework and drawing upon established guidelines [63,64], tools were developed to assess direct and indirect measures of attitudes, subjective norms and PBC, and intention to perform green exercise. Evidence was provided for the factorial validity, composite reliability, and parallel-form reliability for each of the three questionnaires. Consistent with previous studies [15,58,59,60,61], our findings support the theoretical structure of the TPB in relation to performing PA. To date though, no other instruments exist that focus explicitly on beliefs about green exercise. As such, the current findings offer a novel suite of measurement instruments that could be used to provide important insight into the role of individuals’ beliefs in green exercise, thereby contributing to the development of theory and effective interventions.

For content validity, salient beliefs were established through an elicitation study (Study 1). Although valuable for development of TPB questionnaires [62,64], this step is often overlooked within PA research [66]. The salient beliefs captured in the elicitation study informed the development of the BAGE-ID and provide confidence that the items reflect the key cognitions about green exercise in the general population. Consistent with previous research, the advantages and likeable features of green exercise were the most prevalent [77,78]. Interestingly, however, weather—as a disadvantage or dislikeable feature of green exercise—was the most reported salient belief. This may be because the temperate oceanic climate of the UK is not conducive to green exercise throughout the year. Similarly, previous research has found that climatic conditions have an important impact on PA levels across different populations [79,80,81].

Evidence was provided for the factorial validity of the three measures across two samples (refinement and validation). Specifically, following modifications in the refinement sample, the BAGE-ID, BAGE, and INT-GE had good model fits and all factors loadings were significant in both the refinement and validation samples. Of note, however, is that the item relating to weather in the BAGE-ID was removed during the modification process. Although weather was identified as dislikeable feature of green exercise in Study 1, climatic conditions were also listed as a facilitating factor. These contrasting views may partly explain why the factor loading of weather on PBC was not as strong as other items in Study 2. Instead—and congruent with previous research [14,23,47]—items relating to the size, facilities, and safety of local green space may be more reflective of PBC. Overall though, the confirmatory factor analyses indicated that the refined questionnaires had a good factorial validity and model fits were comparable to other questionnaires based on the TPB [82,83].

The current findings provide support for using the TPB as a model to explore green exercise, and the proposed relationships between indirect and direct measures of attitudes and subjective norms. Similarly, previous research has successfully employed the TPB to explore the relationship between PA and nature [15,57]. Consistent with Calogiuri and Chroni [15], we found evidence that the motivational processes associated with green exercise can be modelled from beliefs to intentions. Indeed, previous research has demonstrated that TPB factors can predict intention to engage in outdoor recreation programmes [59] and to visit state parks [58]. The current findings extend the literature by demonstrating that both indirect and direct measures of attitudes, subjective norms, and PBC do predict intention to perform green exercise. Beyond the empirical support for the TPB, these findings could underpin the development of evidence-based interventions to promote intention to perform green exercise.

Previous research has made an important contribution in demonstrating that green exercise has important psychological and health benefits [30,37], and that individuals’ beliefs can facilitate the benefits of PA [8,9]. The questionnaires developed in this study complement this research and provide tools to explore the role of beliefs in green exercise behaviours and outcomes. The consistent use of the three questionnaires will facilitate attempts to synthesise research findings and enable researchers to address theoretically interesting questions, such as which beliefs are the most salient predictors of green exercise behaviours and under what conditions? In total, the questionnaires contain 39 items, all written in the English language with scaled multiple-choice responses, and can be completed in less than 15 min. As such, researchers and health professionals could utilise the tools to assess beliefs in a variety of settings, including field studies, laboratory-based experiments, and applied interventions.

Key strengths of the present research were the use of an elicitation study, the development of questionnaires that assess both indirect and direct measures of attitudes, subjective norms and PBC about green exercise, and the ability to replicate the observed factor structures in two samples. According to the assessment criteria for TPB questionnaire development [64], such steps would enable the current study to achieve Grade A. Despite these strengths, some limitations should be noted. With regards to methodology, the randomisation of participants to the refinement and validation samples in Study 2 led to significant between-group differences for age and gender. In the future, it might be useful to consider stratified random sampling to control for demographics prior to doing confirmatory factor analyses. The correlational nature of Study 2 also limits the ability to infer causality in the relationships between indirect, direct, and intention measures. Further, although the findings demonstrate that beliefs predict the intention to perform green exercise, the relationship with subsequent behaviour was not explored. Future research should therefore explore whether intentions mediate the relationship between beliefs and green exercise behaviour.

## 5. Conclusions

The current article reported the development of three instruments that have great relevance for green exercise research. Although further research is warranted on different samples and using different research designs, the current studies have provided promising initial evidence of the validity and reliability of all three instruments to assess thoughts towards green exercise. For the first time, researchers and health professionals now have the tools to assess the role of beliefs on green exercise behaviours and associated outcomes. Although the questionnaires are presented separately, researchers are encouraged to select the one(s) most relevant for their research. Hopefully, the instruments will contribute to a better understanding of individuals’ beliefs about green exercise, how these influence behaviour, and ultimately to the development of behaviour change interventions designed to promote the use of local green space, and facilitate the psychological and physical outcomes of green exercise.

## Figures and Tables

**Table 1:** Descriptive statistics for the salient beliefs elicited in Study 1.

Advantages and Likes	Total Beliefs	M ± SD	Disadvantages and Dislikes	Total Beliefs	M ± SD	Approve	Total Beliefs	M ± SD
	198	4.9 ± 2.2		133	3.4 ± 2.1		63	1.6 ± 1.0
Fresh Air	32 (16%)		Poor weather	61 (46%)		Family	19 (30%)	
Positive Affect	31 (16%)		Lack of equipment/facilities	17 (13%)		Friends	15 (24%)	
Health and Fitness	29 (15%)		Safety concerns	15 (11%)		Health Professionals	10 (16%)	
Change of Scenery	23 (12%)		Time consuming	11 (10%)		Sports clubs/Trainers	5 (8%)	
Social	20 (10%)		Lack of available green space	10 (7%)		Environmental Groups	5 (8%)	
Openness/Freedom	13 (7%)		Pollution Levels	6 (4%)		Other	5 (8%)	
Costs/Resources	13 (7%)		Lack of privacy	5 (4%)		Employers/Colleagues	4 (6%)	
Nature/Environment	10 (5%)		Cannot find people to do it with	3 (2%)				
Type of activity	8 (4%)		Lack of motivation	3 (2%)				
Access/Availability	8 (4%)		Other	3 (2%)				
Other	6 (3%)							
Weather/Climate	5 (3%)							
**Disapprove**	**11**	**0.3 ± 0.5**	**Easy**	**65**	**1.6 ± 1.2**	**Difficult**	**68**	**1.7 ± 1.2**
Gym Users	5 (42%)		Access/Availability	16 (24%)		Weather	20 (29%)	
Other	4 (33%)		Weather	14 (21%)		Time	20 (29%)	
Gym Companies	3 (25%)		Facilities/Equipment	11 (17%)		Access/Availability	10 (15%)	
			Free time	9 (14%)		Facilities/Equipment	8 (12%)	
			Other	6 (9%)		Activity Groups	5 (7%)	
			Transport	5 (8%)		Lack of motivation	3 (4%)	
			Organised Activity	5 (8%)		Other	2 (3%)	

M ± SD = mean ± standard deviation of beliefs elicited per person. Advantages and Likes = features of green exercise that are perceived favourably. Disadvantages and Dislikes = features of green exercise that are perceived unfavourably. Approve = individuals who would approve of green exercise. Disapprove = individuals who would disapprove of green exercise. Easy = features that would make green exercise easy to do. Difficult = features that would make green exercise difficult to do.

**Table 2:** Fit statistics and factor loadings of single-factor models of the indirect beliefs about green exercise (BAGE-ID).

Factor and Items	Factor Loadings	BSχ^2^	*df*	*p*(BSχ^2^)	CFI	TLI	RMSEA
**Indirect Measure of Attitudes**		**10.84**	**9**	**0.13**	**0.98**	**0.97**	**0.06**
When I do Green Exercise, I feel like I am getting fresh air	0.55						
When I do Green Exercise, I feel better about myself afterwards	0.85						
Green Exercise is good for my health	0.60						
Green Exercise is good for my fitness	0.60						
Doing Green Exercise helps me feel positive about myself	0.82						
Green Exercise is enjoyable	0.60						
**Indirect Measure of Subjective Norms**		**6.23**	**5**	**0.05**	**0.94**	**0.89**	**0.08**
Health professionals would (………...) of me doing Green Exercise	0.31						
My friends think I should do Green Exercise	0.64						
My family think I should do Green Exercise	0.83						
My peers do Green Exercise	0.48						
Current or potential employers would approve of me doing Green Exercise	0.15						
**Indirect Measure of Perceived Behavioural Control**		**6.73**	**5**	**0.06**	**0.91**	**0.82**	**0.88**
The weather influences my decision to do Green Exercise	0.02						
The amount of free time I have influences my decision to do Green Exercise	−0.08						
The amount of green space in my local area influences my decision to do Green Exercise	0.70						
The facilities at my local green space influence my decision to do Green Exercise	0.67						
Safety at my local green space influences my decision to do Green Exercise	0.48						

*n* = 253. BSχ^2^ = Bollen–Stine chi-squared. CFI = comparative fit index. TLI = Tucker–Lewis Index. RMSEA = root mean square error of approximation. All items were scored from *Strongly Disagree* to *Strongly Agree*.

**Table 3:** Fit statistics for two-factor and three-factor model of the indirect beliefs about green exercise (BAGE-ID).

Factor	BSχ^2^	*df*	*p*(BSχ^2^)	CFI	TLI	RMSEA
Attitudes × Subjective Norms	28.83	26	0.03	0.96	0.95	0.06
Attitudes × Perceived Behavioural Control	32.60	26	0.00	0.93	0.90	0.09
Subjective Norms × Perceived Behavioural Control	7.55	8	0.15	0.98	0.97	0.04
Three-factors	44.91	41	0.09	0.97	0.96	0.05

*n* = 253. BSχ^2^ = Bollen–Stine chi-squared. CFI = comparative fit index. TLI = Tucker–Lewis Index. RMSEA = root mean square error of approximation.

**Table 4:** Descriptive statistics, measurement error variances, factor loadings and composite reliabilities for the indirect beliefs about green exercise (BAGE-ID).

Items	Refinement Group (*n* = 253)				Validation Group (*n* = 230)			
	Var(e)	Factor Loadings			Var(e)	Factor Loadings		
		ATT	SUB	PBC		ATT	SUB	PBC
When I do Green Exercise, I feel like I am getting fresh air	0.61	0.63			0.72	0.53		
When I do Green Exercise, I feel better about myself afterwards	0.37	0.79			0.24	0.87		
Green Exercise is good for my fitness	0.47	0.72			0.69	0.56		
Doing Green Exercise helps me feel positive about myself	0.49	0.72			0.31	0.83		
Green Exercise is enjoyable	0.58	0.65			0.65	0.59		
My friends think I should do Green Exercise	0.43		0.75		0.61		0.62	
My family think I should do Green Exercise	0.63		0.61		0.25		0.87	
My peers do Green Exercise	0.82		0.43		0.79		0.46	
The amount of green space in my local area influences my decision to do Green Exercise	0.56			0.66	0.56			0.66
The facilities at my local green space influence my decision to do Green Exercise	0.73			0.52	0.50			0.71
Safety at my local green space influences my decision to do Green Exercise	0.61			0.62	0.77			0.48
Mean response within factors (standard deviation)		15.65 ± 4.66	2.89 ± 3.92	2.31 ± 4.39		14.28 ± 5.10	2.62 ± 3.87	3.31 ± 3.76
Composite Reliability		0.83	0.63	0.63		0.81	0.70	0.65

ATT = Attitudes. SUB = Subjective Norms. PBC = Perceived Behavioural Control. Var(e) = Measurement Error Variance. All items were scored from *Strongly Disagree* to *Strongly Agree*.

**Table 5:** Fit statistics and factor loadings of single-factor models of the beliefs about green exercise (BAGE).

Factor and Items	Factor Loadings	BSχ^2^	*df*	*p*(BSχ^2^)	CFI	TLI	RMSEA
**Attitudes**		**17.34**	**9**	**0.02**	**0.91**	**0.85**	**0.14**
Doing Green Exercise as part of my weekly physical activity is…							
(*Healthy* to *Unhealthy*)	0.54						
(*Bad* to *Good*)	0.62						
(*Pleasant* to *Unpleasant*)	0.76						
(*Boring* to *Fun*)	0.63						
(*Enjoyable* to *Unenjoyable*)	0.69						
(*Beneficial* to *Harmful*)	0.73						
**Subjective Norms**		**5.58**	**5**	**0.15**	**0.95**	**0.90**	**0.06**
Most people who are important to me believe I should do Green Exercise	0.44						
People often ask me to do Green Exercise with them	0.57						
It is expected of me to do Green Exercise	0.36						
I feel under social pressure to do Green Exercise	0.17						
People that are similar to me do Green Exercise	0.56						
**Perceived Behavioural Control**		**4.61**	**5**	**0.07**	**0.89**	**0.77**	**0.06**
I am confident I could do Green Exercise if I wanted to	0.56						
The decision to do Green Exercise is beyond my control	0.30						
Doing Green Exercise is… (*Very Difficult* to Very Easy)	0.44						
Whether I do Green Exercise or not is entirely up to me	0.21						
I choose when and where I do Green Exercise	0.35						

*n* = 253. BSχ^2^ = Bollen–Stine chi-squared. CFI = comparative fit index. TLI = Tucker–Lewis Index. RMSEA = root mean square error of approximation. All items were scored from *Strongly Disagree* to *Strongly Agree*, unless stated otherwise.

**Table 6:** Fit statistics for the two-factor and three-factor model of the beliefs about green exercise (BAGE).

Factor	BSχ^2^	*df*	*p*(BSχ^2^)	CFI	TLI	RMSEA
Attitudes × Subjective Norms	22.72	19	0.20	0.98	0.97	0.05
Attitudes × Perceived Behavioural Control	27.52	26	0.31	0.99	0.99	0.03
Subjective Norms × Perceived Behavioural Control	13.40	13	0.14	0.95	0.92	0.04
Three-factors	53.35	51	0.02	0.98	0.97	0.03

*n* = 253. BSχ^2^ = Bollen–Stine chi-squared. CFI = comparative fit index. TLI = Tucker–Lewis Index. RMSEA = root mean square error of approximation.

**Table 7:** Descriptive statistics, measure error variances, factor loadings and composite reliabilities for the beliefs about green exercise (BAGE).

Items	Refinement Group (*n* = 253)				Validation Group (*n* = 230)			
	Var(e)	Factor Loadings			Var(e)	Factor Loadings		
		ATT	SUB	PBC		ATT	SUB	PBC
Doing Green Exercise as part of my weekly physical activity is…								
(*Bad* to *Good*)	0.62	0.62			0.39	0.78		
(*Pleasant* to *Unpleasant*)	0.42	0.76			0.34	0.81		
(*Boring* to *Fun*)	0.55	0.67			0.39	0.78		
(*Enjoyable* to *Unenjoyable*)	0.48	0.72			0.42	0.76		
(*Beneficial* to *Harmful*)	0.56	0.66			0.52	0.69		
Most people who are important to me believe I should do Green Exercise	0.80		0.45		0.83		0.41	
People often ask me to do Green Exercise with them	0.59		0.64		0.48		0.72	
People that are similar to me do Green Exercise	0.76		0.49		0.60		0.63	
I am confident I could do Green Exercise if I wanted to	0.80			0.45	0.67			0.57
The decision to do Green Exercise is beyond my control	0.93			0.27	0.83			0.41
Doing Green Exercise is… (*Very Difficult* to *Very Easy*)	0.63			0.61	0.64			0.60
I choose when and where I do Green Exercise	0.93			0.27	0.80			0.44
Mean response within factors (standard deviation)		6.41 ± 0.74	4.35 ± 0.99	5.97 ± 0.73		6.11 ± 0.94	4.31 ± 0.99	5.63 ± 0.86
Composite Reliability		0.82	0.54	0.44		0.88	0.62	0.58

ATT = Attitudes. SUB = Subjective Norms. PBC = Perceived Behavioural Control. Var(e) = Measurement Error Variance. All response scales ranged from *Strongly Disagree* to *Strongly Agree*, unless stated otherwise.

**Table 8:** Fit statistics and factor loadings of the intentions to perform green exercise (INT-GE).

Factor and Items	Factor Loadings	BSχ^2^	*df*	*p*(BSχ^2^)	CFI	TLI	RMSEA
Intention		5.79	5	0.66	1.00	1.01	0.00
I expect to do Green Exercise	0.71						
I want to do Green Exercise	0.70						
The likelihood of me doing Green Exercise is…(*Very Unlikely* to *Very Likely*)	0.87						
I plan to do Green Exercise	0.81						
I intend to do Green Exercise	0.84						

*n* = 253. BSχ^2^ = Bollen–Stine chi-squared. CFI = comparative fit index. TLI = Tucker–Lewis Index. RMSEA = root mean square error of approximation. All items were scored from *Strongly Disagree* to *Strongly Agree*, unless stated otherwise.

## References

[1] World Health Organisation Prevalence of insufficient physical activity. http://www.who.int/gho/ncd/risk_factors/physical_activity_text/en/.

[2] Ding D., Lawson K.D., Kolbe-Alexander T.L., Finkelstein E.A., Katzmarzyk P.T., van Mechelen W., Pratt M. (2016). The economic burden of physical inactivity: A global analysis of major non-communicable diseases. Lancet.

[3] Academy of Medical Royal Colleges (2015). Exercise: The Micracle Cure and Role of the Doctor in Promoting It.

[4] National Health Service Benefits of Exercise. http://www.nhs.uk/Livewell/fitness/Pages/whybeactive.aspx.

[5] Haskell W.L., Lee I.M., Pate R.R., Powell K.E., Blair S.N., Franklin B.A., Macera C.A., Heath G.W., Thompson P.D., Bauman A. (2007). Physical activity and public health. Updated recommendation for adults from the american college of sports medicine and the American Heart Association. Circulation.

[6] Downs D.S., Hausenblas H.A. (2005). The theories of reasoned action and planned behaviour applied to exercise: A meta-analytic update. J. of Phys. Act. Health.

[7] Hagger M.S., Chatzisarantis N.L., Biddle S.J. (2002). A meta-analytic review of the theories of reasoned action and planned behavior in physical activity: Predictive validity and the contribution of additional variables. J. Sport Exerc. Psychol.

[8] Darker C.D., French D.P., Eves F.F., Sniehotta F.F. (2010). An intervention to promote walking amongst the general population based on an ‘extended’ theory of planned behaviour: A waiting list randomised controlled trial. Psychol. Health.

[9] Parrott M.W., Tennant L.K., Olejnik S., Poudevigne M.S. (2008). Theory of planned behavior: Implications for an email-based physical activity intervention. Psychol. Sport Exerc..

[10] Pretty J., Griffin M., Sellens M. (2003). Green exercise: Complementary roles of nature, exercise and diet in physical and emotional well-being and implications for public health policy. CES Occasional Paper 2003-1.

[11] Fromel K., Kudlacek M., Groffik D., Svozil Z., Simunek A., Garbaciak W. (2017). Promoting healthy lifestyle and well-being in adolescents through outdoor physical activity. Int. J. Environ. Res. Public Health.

[12] Calogiuri G., Patil G.G., Aamodt G. (2016). Is green exercise for all? A descriptive study of green exercise habits and promoting factors in adult norwegians. Int. J. Environ. Res. Public Health.

[13] Brymer E., Davids K. (2016). Designing environments to enhance physical and psychological benefits of physical activity: A multidisciplinary perspective. Sports Med..

[14] Flowers E.P., Freeman P., Gladwell V.F. (2016). A cross-sectional study examining predictors of visit frequency to local green space and the impact this has on physical activity levels. BMC Public Health.

[15] Calogiuri G., Chroni S. (2014). The impact of the natural environment on the promotion of active living: An integrative systematic review. BMC Public Health.

[16] Shanahan D.F., Bush R., Gaston K.J., Lin B.B., Dean J., Barber E., Fuller R.A. (2016). Health benefits from nature experiences depend on dose. Sci. Rep..

[17] Gidlow C.J., Randall J., Gillman J., Smith G.R., Jones M.V. (2016). Natural environments and chronic stress measured by hair cortisol. Landsc. Urban Plan..

[18] Pope D., Tisdall R., Middleton J., Verma A., Van Ameijden E., Birt C., Bruce N. (2015). Quality of and access to green space in relation to psychological distress: Results from a population-based cross-sectional study as part of the euro-urhis 2 project. Eur. J. Public Health.

[19] Sugiyama T., Leslie E., Giles-Corti B., Owen N. (2008). Associations of neighbourhood greenness with physical and mental health: Do walking, social coherence and local social interaction explain the relationships?. J. Epidemiol. Community Health.

[20] Maas J., Verheij R.A., Spreeuwenberg P., Groenewegen P.P. (2008). Physical activity as a possible mechanism behind the relationship between green space and health: A multilevel analysis. BMC Public Health.

[21] Mytton O.T., Townsend N., Rutter H., Foster C. (2012). Green space and physical activity: An observational study using health survey for england data. Health Place.

[22] Veitch J., Salmon J., Parker K., Bangay S., Deforche B., Timperio A. (2016). Adolescents’ ratings of features of parks that encourage park visitation and physical activity. Int. J. Behav. Nutr. Phys. Act..

[23] Kaczynski A.T., Besenyi G.M., Stanis S.A., Koohsari M.J., Oestman K.B., Bergstrom R., Potwarka L.R., Reis R.S. (2014). Are park proximity and park features related to park use and park-based physical activity among adults? Variations by multiple socio-demographic characteristics. Int. J. Behav.Nutr. Phys. Act..

[24] Nordh H., Hartig T., Hagerhall C.M., Fry G. (2009). Components of small urban parks that predict the possibility for restoration. Urban For. Urban Green..

[25] Henderson-Wilson C., Sia K.L., Veitch J., Staiger P.K., Davidson P., Nicholls P. (2017). Perceived health benefits and willingness to pay for parks by park users: Quantitative and qualitative research. Int. J. Environ. Res. Public Health.

[26] World Health Organisation Europe (2016). Urban Green Spaces and Health—A Review of Evidence.

[27] Refshauge A.D., Stigsdotter U.K., Cosco N.G. (2012). Adults’ motivation for bringing their children to park playgrounds. Urban For. Urban Green..

[28] Irvine K.N., Warber S.L., Devine-Wright P., Gaston K.J. (2013). Understanding urban green space as a health resource: A qualitative comparison of visit motivation and derived effects among park users in Sheffield, UK. Int. J. Environ. Res. Public Health.

[29] Rogerson M., Gladwell V.F., Gallagher D.J., Barton J.L. (2016). Influences of green outdoors versus indoors environmental settings on psychological and social outcomes of controlled exercise. Int. J. Environ. Res. Public Health.

[30] Thompson Coon J., Boddy K., Stein K., Whear R., Barton J., Depledge M.H. (2011). Does participating in physical activity in outdoor natural environments have a greater effect on physical and mental wellbeing than physical activity indoors? A systematic review. Environ. Sci. Technol..

[31] Hug S.M., Hartig T., Hansmann R., Seeland K., Hornung R. (2009). Restorative qualities of indoor and outdoor exercise settings as predictors of exercise frequency. Health Place.

[32] Kerr J.H., Fujiyama H., Sugano A., Okamura T., Chang M., Onouha F. (2006). Psychological responses to exercising in laboratory and natural environments. Psychol. Sport Exerc..

[33] Bodin M., Hartig T. (2003). Does the outdoor environment matter for psychological restoration gained through running?. Psychol. Sport Exerc..

[34] Bowler D.E., Buyung-Ali L.M., Knight T.M., Pullin A.S. (2010). A systematic review of evidence for the added benefits to health of exposure to natural environments. BMC Public Health.

[35] Gidlow C.J., Jones M.V., Hurst G., Masterson D., Clark-Carter D., Tarvainen M.P., Smith G., Nieuwenhuijsen M. (2016). Where to put your best foot forward: Psycho-physiological responses to walking in natural and urban environments. J. Environ. Psychol..

[36] Brown D.K., Barton J.L., Pretty J., Gladwell V.F. (2014). Walks4work: Assessing the role of the natural environment in a workplace physical activity intervention. Scand. J. Work Environ. Health.

[37] Gladwell V.F., Brown D.K., Wood C., Sandercock G.R., Barton J.L. (2013). The great outdoors: How a green exercise environment can benefit all. Extrem. Physiol. Med..

[38] Triguero-Mas M., Dadvand P., Cirach M., Martinez D., Medina A., Mompart A., Basagana X., Grazuleviciene R., Nieuwenhuijsen M.J. (2015). Natural outdoor environments and mental and physical health: Relationships and mechanisms. Environ. Int..

[39] Aspinall P., Mavros P., Coyne R., Roe J. (2015). The urban brain: Analysing outdoor physical activity with mobile eeg. Br. J. Sports Med..

[40] Mackay G.J., Neill J.T. (2010). The effect of “green exercise” on state anxiety and the role of exercise duration, intensity, and greenness: A quasi-experimental study. Psychol. Sport Exerc..

[41] Akers A., Barton J., Cossey R., Gainsford P., Griffin M., Micklewright D. (2012). Visual color perception in green exercise: Positive effects on mood and perceived exertion. Environ. Sci. Technol..

[42] Barton J., Pretty J. (2010). What is the best dose of nature and green exercise lor improving mental health? A multi-study analysis. Environ. Sci. Technol..

[43] Crust L., Henderson H., Middleton G. (2013). The acute effects of urban green and countryside walking on psychological health: A field-based study of green exercise. Int. J. Sport Psychol..

[44] Pretty J., Peacock J., Hine R., Sellens M., South N., Griffin M. (2007). Green exercise in the uk countryside: Effects on health and psychological well-being, and implications for policy and planning. J. Environ. Plan. Manag..

[45] Wood C., Angus C., Pretty J., Sandercock G., Barton J. (2013). A randomised control trial of physical activity in a perceived environment on self-esteem and mood in UK adolescents. Int. J. Environ. Health Res..

[46] Lackey K.J., Kaczynski A.T. (2009). Correspondence of perceived vs. Objective proximity to parks and their relationship to park-based physical activity. Int. J. Behav. Nutr. Phys. Act..

[47] Bai H., Wilhelm Stanis S.A., Kaczynski A.T., Besenyi G.M. (2013). Perceptions of neighborhood park quality: Associations with physical activity and body mass index. Ann. Behav. Med..

[48] Leslie E., Sugiyama T., Ierodiaconou D., Kremer P. (2010). Perceived and objectively measured greenness of neighbourhoods: Are they measuring the same thing?. Landsc. Urban Plan..

[49] Dunlap R.E., Van Liere K.D. (1978). The “new environmental paradigm”. J. Environ. Educ..

[50] Nisbet E.K., Zelenski J.M., Murphy S.A. (2008). The nature relatedness scale: Linking individuals’ connection with nature to environmental concern and behavior. Environ. Behav..

[51] Plotnikoff R.C., Lubans D.R., Costigan S.A., Trinh L., Spence J.C., Downs S., McCargar L. (2011). A test of the theory of planned behavior to explain physical activity in a large population sample of adolescents from Alberta, Canada. J. Adolesc. Health.

[52] Wankel L.M., Mummery W.K. (1993). Using national survey data incorporating the theory of planned behavior: Implications for social marketing strategies in physical activity. J. Appl. Sport Psychol..

[53] Ajzen I., Fishbein M. (1980). Understanding Attitudes and Predicting Social Behavior.

[54] Francis J., Johnston M., Eccles M.P., Grimshaw J.M., Kaner E.F.S. (2004). Measurement Issues in the Theory of Planned Behaviour.

[55] Ajzen I., Driver B.L. (1991). Prediction of leisure participation from behavioral, normative, and control beliefs: An application of the theory of planned behavior. Leis. Sci..

[56] Biddle S.J.H., Mutrie N., Gorely T. (2015). Psychology of Physical Activity: Determinants, Well-Being and Interventions.

[57] Nelson N.M., Wright A., Lowry R.G., Mutrie N. (2008). Where is the theoretical basis for understanding and measuring the environment for physical activity?. Environ.Health Insights.

[58] Shrestha S.K., Burns R.C. An assessment of the efficacy of the theory of planned behaviour to predict intentions to visit state parks. Proceedings of the George Wright Society Biennial Conference on Parks, Protected Areas, and Cultural Sites.

[59] Kouthouris C.H., Spontis A. (2005). Outdoor recreation participation: An application of the theory of planned behaviour. Sports J..

[60] Rhodes R.E., Brown S.G., McIntyre C.A. (2006). Integrating the perceived neighborhood environment and the theory of planned behaviour when predicting walking. Am. J. Health Behav..

[61] Middlestadt S.E., Anderson A., Ramos W.D. (2015). Beliefs about using an outdoor pool: Understanding perceptions of place in the context of a recreational environment to improve health. Health Place.

[62] Ajzen I. Constructing a tpb questionnaire: Conceptual and methodological considerations. http://people.umass.edu/aizen/pdf/tpb.measurement.pdf.

[63] Francis J., Eccles M.P., Johnston M., Walker A.E., Grimshaw J.M., Foy R., Kaner E.F.S., Smith L., Bonetti D. (2004). Constructing Questionnaires Based on the Theory of Planned Behaviour: A Manual for Health Services Researchers.

[64] Oluka O.C., Nie S., Sun Y. (2014). Quality assessment of TPB-based questionnaires: A systematic review. PLoS ONE.

[65] Godin G., Kok G. (1996). The theory of planned behavior: A review of its applications to health-related behaviors. Am. J. Health Promot..

[66] Downs D.S., Hausenblas H.A. (2005). Elicitation studies and the theory of planned behavior: A systematic review of exercise beliefs. Psychol. Sport Exerc..

[67] Joreskog K.G., Bollen K.A., Long J.S. (1993). Testing structural equation models. Testing Structural Equation Models.

[68] Enders C.K. (2002). Applying the bollen-stine bootstrap for goodness-of-fit measures to structural equation models with missing data. Multivar. Behav. Res..

[69] Hu L.t., Bentler P.M. (1999). Cutoff criteria for fit indexes in covariance structure analysis: Conventional criteria versus new alternatives. Struct. Equ. Model. Multidiscip. J..

[70] Jackson D.L., Gillaspy J.A., Purc-Stephenson R. (2009). Reporting practices in confirmatory factor analysis: An overview and some recommendations. Psychol. Methods.

[71] Marsh H.W., Hau K.T., Wen Z. (2004). In search of golden rules: Comment on hypothesis-testing approaches to setting cutoff values for fit indexes and dangers in overgeneralizing hu and bentler’s (1999) findings. Struct. Equ. Model..

[72] Jöreskog K.G., Sörbom D. (1996). Lisrel 8: User’s Reference Guide.

[73] Stevens J.P. (2009). Applied Multivariate Statistics for the Social Sciences.

[74] Fornell C., Larcker D.F. (1981). Evaluating structural equation models with unobservable variables and measurement error. J. Mark. Res..

[75] Office for National Statistics UK Labour Market: January 2017. https://www.ons.gov.uk/employmentandlabourmarket/peopleinwork/employmentandemployeetypes/bulletins/uklabourmarket/jan2017#employment.

[76] Blunch N. (2012). Introduction to Structural Equation Modeling Using IBM Spss Statistics and Amos.

[77] Sutton S., French D.P., Hennings S.J., Mitchell J., Wareham N.J., Griffin S., Hardeman W., Kinmonth A.L. (2003). Eliciting salient beliefs in research on the theory of planned behaviour: The effect of question wording. Curr. Psychol. Dev. Learn. Personal. Soc..

[78] Darker C.D., French D.P., Longdon S., Morris K., Eves F.F. (2007). Are beliefs elicited biased by question order? A theory of planned behaviour belief elicitation study about walking in the UK general population. Br. J. Health Psychol..

[79] Remmers T., Thijs C., Timperio A., Salmon J., Veitch J., Kremers S.P., Ridgers N.D. (2017). Daily weather and children’s physical activity patterns. Med. Sci. Sports Exerc..

[80] Tucker P., Gilliland J. (2007). The effect of season and weather on physical activity: A systematic review. Public Health.

[81] Witham M.D., Donnan P.T., Vadiveloo T., Sniehotta F.F., Crombie I.K., Feng Z., McMurdo M.E. (2014). Association of day length and weather conditions with physical activity levels in older community dwelling people. PLoS ONE.

[82] González S.T., López M.C.N., Marcos Y.Q., Rodríguez-Marín J. (2013). Development and validation of the theory of planned behavior questionnaire in physical activity. Span. J. Psychol..

[83] Fen Y.S., Sabaruddin N.A. (2009). An extended model of theory of planned behaviour in predicting exercise intention. Int. Bus. Res..

